# American Medical Association

**Published:** 1879-05

**Authors:** 


### AMERICAN MEDICAL ASSOCIATION.

TUESDAY'S PROCEEDINGS.

ATLANTA, May 6th, 1879.

The Association was called to order by President Parvin, and the exercises were opened with prayer from Rev. Dr. Gwinn.

Next in order was the welcome address, by Dr. J. P. Logan, of Atlanta, Ga. The speaker was introduced by the President, and proceeded to make some very appropriate and eloquent remarks of welcome to the members of the Association.

Report of Committee on Credentials being next in order, Dr. Logan, as chairman, remarked that the registration was in the hands of the permanent Secretary, Dr. Atkinson, who proceeded to read the roll of the accredited members present.

The Secretary then read the programme of receptions, as offered by the citizens of Atlanta to the Association.

Dr. Logan stated an invitation of the citizens of Augusta, to visit that city on adjournment of the Association.

Dr. Campbell expressed an earnest hope that the invitation to visit his city would be accepted, as he was satisfied every effort would be made to have members enjoy the trip.

Next in order was the reading of notes from absentees. Notes were received from Drs. Bowditch, Hutchinson, and Storer, stating that they were Providentially compelled to be absent.

The paper of Dr. Hutchinson on the treatment of aneurism was not read, on account of his absence.

The President then delivered the annual address, which was replete with philosophy, eloquence and logic.

Dr. Brodie moved to return thanks for the address, and to request a copy for publication, which motion was unanimously carried by a rising vote.

Dr. Logan moved that the ex-Presidents of the Association who were present be invited to take seats on the stage.

The President invited Drs. Gross, Richardson, Davis and Storer. The gentlemen advanced amid applause, and took the seats assigned them.

The next business was a call for voluntary papers.

Dr. Sayre presented a very interesting paper, which, on motion, was referred to section on Surgery.

Dr. Seguin presented a paper suggesting the adoption of the metric system, by the Association, with a resolution to that effect.

Dr. Keller, of New York, moved that the consideration of Dr. Seguin's resolution be deferred until Thursday.

Dr. Brodie moved to refer the resolution to a Committee, to report at next annual meeting.

Dr. Keller would not accept the amendment.

The motion to defer till Thursday was carried.

Dr. Bell made a motion to consolidate Sections four and five, to be called Section four. Carried.

Dr. Keller moved that the Committee on Nomination be made up of members present only.

Dr. Davis rose to a point of order, and suggested that amendments should not be considered so soon, as the order of business placed their consideration later. Moved that the Association adjourn till Wednesday morning, at 9.30. Carried.

The following is the programme for the session of the American Medical Association, as announced in the first day's meeting:

The thirtieth annual meeting of the American Medical Association, will be held in DeGive's Opera House, corner of Marietta and Forsyth streets, commencing to-day, May 6, at 11 o'clock A.M.

11 A.M. The President, Dr. Theophilus Parkins, of Indianapolis, will call the Association to order.

Prayer by the Rev. D. W. Gwin, D.D.

Address of welcome, by Dr. Joseph P. Logan, Chairman of Committee of Arrangements.

Report of Committee of Arrangements on credentials of members.

Election of members by invitations, and of permanent members by the recommendation of the Committee of Arrangements.

Reading of notes from absentees.

12 M. Annual Address of the President. Call for reports from special Committees and volunteer papers, and their reference to Sections.

Report on the adoption of the metric system by the Association, by Dr. E. Seguin, of New York.

1 P.M. Adjournment.

3 P.M. Sections: Section I. Practical Medicine, Materia Medica and Physiology. Chairman, Dr. Thos. F. Rochester, of Buffalo, New York; Secretary, Dr. W. D. Glascow, of St. Louis, Missouri.

Experience of consumptives in Colorado and some other of the æro-hygiencies of elevation above the sea, with conclusions; by Dr. Charles Denison, of Denver, Colorado.

Clinical and Metereological Records; by Dr. N. S. Davis, of Illinois.

Section II.—Obstetrics and Diseases of Women and Children, Chairman, Dr. E. S. Lewis, of New Orleans, Louisiana; Secretary, (vacant.)

Tubo-ovarian Gestation (case,) operation fifth month; death; by Dr. Robert Battey, of Georgia.

Electrolysis of Fibroids; by Dr. E. Cutter, of Massachusetts.

Dysmenorrhœa; by Dr. W. H. Byford, of Illinois.

Section III.—Surgery and Anatomy, Chairman, Dr. Moses Gunn, of Chicago, Illinois; Secretary, Dr. J. R. West, of Richmond, Indiana.

Aspiration of the Knee Join; by Dr. H. O. Marcy, of Massachusetts.

On Deformities of the Face and Hands, occasioned by Cicatricial Contraction following a Burn; by Dr. A. C. Post, of New York.

New surgical needle, curved and sprung clamp at point; new apparatus for treating fracture of the clavicle, with case; new method of reducing dislocation of elbow-joint, with cases; by Dr. E. B. Turnipseed, of South Carolina.

Section IV. Medical jurisprudence, chemistry and physchology. Chairman (deceased); Secretary, Dr. L. M. Eastman, of Baltimore, Md.

Section V. State medicine and public hygiene. Chairman, Dr. John S. Billings, of Washington, D. C.; Secretary, Dr. J. T. Reeve, Appleton, Wis.

The regulation of medical practice by State Boards of Health, as exemplified in Illinois; by Dr. H. A. Johnson, of Illinois.

State medical societies and State medicine; by Dr. S. E. Chaille of Louisiana.

Section VI. Opthalmology, otology, laryngology. Chairman, Dr. H. Knapp, of New York; Secretary, Dr. X. C. Scott, Cleveland, O.

Cataract extraction; by Dr. H. Knapp, of New York.

An operation for the radical cure of cystoid cicatrix; by D. S. Reynolds, of Kentucky.

---

SECOND DAY—WEDNESDAY, MAY 7.

ATLANTA, GA., May 7, 1879.

Association called to order by Vice-President Murphy.

Some sensation was produced by motions opposed to the abolition of duties on quinine. The motion to table was carried.

Dr. Robert moved that Congress be again asked to remove the duties on the drug. Carried.

In the regular order of business Dr. Rochester, of New York, proceeded to address the Association. He was listened to with marked attention, and very often applauded.

Dr. Atchison moved that the address be referred to the section of pratical medicine.

Amendment to refer portions to another section was lost, when the original motion was carried.

The President announced the address of Dr. Billings in order, when Dr. J. J. Woodward, U. S. A., on account of illness of the author, proceeded to read the address.

A motion to refer the address to the appropriate section was carried.

General F. A. Walker, on motion, was invited to a seat on the stage, and was introduced by the President amid applause.

Next in order was amendment proposing changes in organization, but by permission Dr. Davis, Chairman of a special committee appointed last year to act on the suggestions of Dr. Richardson, in his annual address presented the report. On this Committee were appointed Drs. Davis, Gross and Toner.

It was moved that the report be received and, under the rule, referred over till next annual meeting.

The Secretary read an additional list of members who arrived since yesterday.

Dr. Davis moved that the names proposed for membership by invitation, be referred to the Committee of Arrangements, which motion was carried.

The regular order was then taken up and the amendment that the Committee on Nominations should hereafter select the nominees only from members present, was moved to be laid on the table. A rising vote was called for and resulted, yeas, 120; nays, 5; so the amendment was laid on the table.

Dr. Hitchcock proposed an amendment last year and upon its being brought up the Secretary read a letter from Dr. Hitchcock explaining its merits.

Dr. Reynolds remarked that as the measure reflected upon the honor of the Association, he moved that it be tabled, which was done.

The amendment by Dr. Caldwell was tabled.

Dr. T. K. Maddox wanted to create a new section on genito-urinary trouble. Motion to lay on the table was lost, when the question was discussed by Doctors Davis, Brown of Texas, and others, and finally referred to the section on surgery on motion of Dr. Pratt.

Dr. Davis, as Chairman of a Committee appointed to revise certain portions of the Code of Ethics, made a report. This was against the practice of certain schools teaching students who are known to have intentions of practicing some exclusive system of medicine.

Dr. Dunster, in a very effective and brilliant speech, exposed the fallacy of the measure, and presented such legal and rational arguments against the suggestions made in the report, that they were hopelessly neutralized.

Dr. Davis replied to Dr. Dunster by stating his position with regard to the report; remarked that the Committee did not necessarily endorse it as their views, but they were forced, by exactions, to report something, and this was the best they could do.

Dr. Pratt made some remarks preliminary to a motion to lay the proposed amendment on the table until next year.

Dr. Brodie moved to lay Dr. Pratt's motion on the table until some discussion was had, which motion was lost by a rising vote, yeas, 72; nays, 122.

The motion to table till next year was then carried.

The roll of States was now called by the Secretary so that delegates from each might choose places to meet and elect members of Nominating Committee.

The Association then adjourned until to-morrow at 9:30 a. m.

---

THIRD DAY—WEDNESDAY, MAY 8TH, 9½ A. M.

Promptly the American Medical Association met in the Opera House, President Parvin in the Chair.

The Secretary announced several communications from the committee on arrangements relative to the banquet of Thursday night, the excursion to Augusta and other points of interest to the members.

Reports were next in order, and the following were offered:

Dr. N. S. Davis, of Chicago, offered a brief but pointed report on ozone, which was referred to the Committee on Publication.

The report on necrology was presented by ex-President J. M. Toner, of Washington, the Chairman of the Committee.

The report on sanitasia for consumptives was to have been presented by Dr. H. I. Bowditch, of Boston, but he was absent and the report was postponed. The report on the catalogue of the National Library by Dr. H. C. Wood, of Pennsylvania, was received. It stated that Congress had been induced at last to publish two of these valuable journals.

Dr. Woodward, of the army, thanked the Association for the warm support it had given his colleague, Dr. Billings, in his efforts to complete a great and necessary work. The report was referred to the Committee on Publication.

Dr. Atkinson, the Secretary, presented the report of the Committee on Publication, which stated the entire work of that committee for the past year. The report was received and disposed of as those above.

The Treasurer's report was next read and referred to the Committee on Publication.

The Librarian's report was also read and referred to the same committee.

The next business in order was the presentation of a paper on State medicine, which Dr. Chaille, of New Orleans, had read in the section of State medicine Tuesday afternoon. The paper took such a philosophical view of the great question that the section ordered it referred to the Association. Dr. Chaille's views are along with the most advanced theories on this subject. He appealed for the thorough establishment of a sound system of State medicine, in which the general Government should regulate without interfering with any of the necessary powers of the State. The paper was referred back to the section where it had been first presented.

The President—The hour has arrived for the address of Dr. Moses Gunn, of Chicago, Chairman of the section on surgery and anatomy. [Applause.]

The Doctor advanced to the edge of the desk and delivered an address worthy of his high position in the profession. Though the paper was long it held the closest attention of the entire Association. The paper treated of pus in the most scientific manner.

The paper was referred to the section on surgery.

NOMINATIONS.

Dr. S. D. Gross, of Philadelphia, announced that the Committee on Nominations was ready to report. The following report was then received through the Secretary of the committee, Dr. Eugene Grissom, of North Carolina.

REPORT ON NOMINATIONS.

The Committee on Nominations presented the following:

President—Dr. Lewis A. Sayre, of New York.

Vice-Presidents—

First—Dr. R. Beverly Cole, of California.

Second—Dr. E. M. Hunt, of New Orleans.

Third—Dr. H. O. Massey, of Massachusetts.

Fourth—Dr. F. Peyer Porcher, of South Carolina.

Treasurer—Dr. R. J. Dunglison, of Pennsylvania.

Librarian—Dr. Wm. Lee, of District of Columbia.

Committee on Library—Dr. Johnson Eliot, of District of Columbia.

Assistant Secretary—Dr. Walter Gillette, of New York.

Next place of meeting—New York.

Time of meeting—Not yet fixed.

Committee on Arrangements—Drs. L. O. Vanderpoel, Stephen Smith, William M Polk, Robert Weir, Charles I. Pardee, A. A. Smith, T. F. Sabine, of New York; Joseph Hutchison, of Brooklyn; M. H. Burton, of Troy, and Dr. Parker, of Poughkeepsie.

Committee on Prize Essays—Not yet appointed.

Committee on Publication—Doctors W. B. Atkinson, T. M. Dugsdole, A. Fricke, S. D. Gross, Caspar Wistar, R. J. Dunglison, of Pennsylvania, and William Lee, of District of Columbia.

The following nominations for Chairmen and Secretaries for sections of 1880 are reported:

1. Practice of medicine, etc.—Dr. J. S. Lynch, of Maryland, Chairman; Dr. W. C. Glasgow, of Missouri, Secretary.

2. Obstetrics, etc—Dr. Albert Smith, of Pennsylvania, Chairman; Dr. Robert Battey, of Georgia, Secretary.

3. Surgery and Anatomy—Dr. W. T. Briggs, of Tennessee, Chairman; Dr. J. Powell Adams, of Minnesota, Secretary.

4. State Medicine, Medical Jurisprudence, etc—Dr. J. F. Hibbard, of Indiana, Chairman; Dr. T. F. Wood, of North Carolina, Secretary.

5. Opthalmology, etc—Dr. Bolling A. Pope, of Louisiana, Chairman; Dr. Eugene Smith, of Michigan, Secretary.

Committee on Necrology—Dr. J. M. Toner, of District of Columbia, Chairman; Drs. R. F. Mitchell, of Alabama, J. P. Wall, of Florida; F. W. Hatch, of California; J. B. Cummings, of Arkansas; C. Denison, of Colorado; G. W. Russell, of Connecticut; J. H. Richards, of Delaware; T. S. Hopkins, of Georgia; J. H. Hollister, of Illinois; G. L Sutton, of Indiana; H. B. Ransom, of Iowa; C. V. Mottrom, of Kansas; Dudley S. Reynolds, of Kentucky; E. A. Lewis, of

Louisiana; E. F. Sanger, of Maine; John Morrison, of Maryland; L. F. Warner, of Massachusetts; G. E. Barney, of Michigan; D. W. Hand, of Minnesota; John Browning, of Mississippi; J. M. Richmond, of Missouri; J. R. Black, of Nebraska; L. S. Hill, of New Hampshire; H. D. Didama, of New York; John Blaine, of New Jersey; T. J. Haywood, jr., of North Carolina; Starling Loving, of Ohio; Frank Woodbury, of Pennsylvania; C. H. Fisher, of Rhode Island; M. Simmons, of South Carolina; J. B. Lindsay, of Tennessee; H. W. Brown, of Texas; O. F. Fassett; of Vermont; L. S. Joynes, of Virginia; R. W. Hazlett, of West Virginia; J. T. Reeves, of Wisconsin; J. J. Woodward, of United States Army; A. L. Guion, of United States Army.

A further report will be made hereafter.

On motion, the report was received and the nominations endorsed.

The next business was the reading of an address by Dr. E. S. Lewis, of New Orleans, chairman of the Section on Obstetrics, etc.

The paper was referred to the Committee on Publication.

The next business was the consideration of Dr. Seguin's report on the metric system. The doctor made a few remarks in support of his resolutions. They are as follows:

*Resolved*, 1. That the American Medical Association adopts the international metric system, and will use it in its transactions.

2. Requests that those who present papers at its future meetings employ this system in their communications, or reprint thereof.

3. Requests the medical boards of the hospitals and dispensaries to adopt the metric system in prescribing and recording cases; and that the faculties of the medical and pharmaceutic schools adopt it in their didactic, clinical or dispensing departments.

4. Requests the physicians familiar with the metric system to help their confreres and the druggists in its application; and the delegates present at this session to work up the acceptance of the metric system by their respective county and State societies.

5. Requests our President to name a Metric Executive Committee, of which he shall be the ex-officio chairman, and whose task will be to give unity and rapidity to this metric movement.

On motion, they were adopted.

Dr. Chaille, of New Orleans, offered a resolution that Congress be petitioned to allow any student of scientific pursuits to import free of duty any one book for his own use. Adopted.

Dr. Brodie, of Detroit, referred to the judical council a query as to the propriety of the use of patent medicines, and a resolution declaring such use against the code of ethics.

Some amendments were laid over to next year.

Association adjourned till to-morrow at 9 o'clock.

---

FOURTH DAY'S PROCEEDINGS—MAY 9, 9½ A.M.

The Convention was called to order by President Parvin.

Communications from the Committee of Arrangements were read.

Reports from all the five Sections were read. These reports contained the subjects on which the sections treated.

A resolution passed by the Section on State Medicine, looking to the thorough organization of State Societies, and prescribing stricter rules for admission into the American Medical Association, were passed.

A communication from the California Medical Association, pressing the necessity of a national quarantine, was read and referred.

Dr. Knapp, of New York, read a paper, which on motion was referred to the Committee on Publication.

Under the order of report of Committee on Prize Essays, the Secretary read the report.

The Committee decided that the prize of one hundred dollars be awarded Dr. Hamilton, of New York, and that the other essay not coming up to the standard proposed by the Association was not recommend for award.

The President appointed the following Committees:

To represent the Association abroad—Drs. Seguin, Yandell, DaCosta, Gunn, Turnbull, Warren and J. T. Hodgson.

Delegates to Canadian Association—Dr. H. Hutchins, Dr. W. Brodie.

On Dr. Chaille's resolution on public hygiene and its regulation by Congress—Drs. Pratt, Davis, Garcelon, Gross and Bell.

The Committee on Nominations, reported their work complete and presented the following:

Time of meeting—First Tuesday in June, 1880. Place—New York.

Committee on prize essays—Drs. Austin Flint, Chairman; Alfred C. Post, Joseph Hutchinson, J. W. S. Gouley, Montrose H. Pallen, all of New York.

Members of section on State Medicine, public Hygiene, etc.—Drs. W. H. Hawkins, Arkansas; Jerome Cochrane, Alabama; W. F. Cherry, California; C. Dennison, Colorado; C. A. Lindley, Connecticut; Wm. Marshall, Delaware; Thos. Antisell, District of Columbia; J. P. Wall, Florida; J. P. Logan, Georgia; S. Brondeis, Kentucky; S. E. Chaille, Louisiana; A. P. Snow, Maine; T. B. Evans, Maryland; H. I. Bowditch, Massachusetts; H. B. Baker, Michigan; C. N. Hewett, Minnesota; Wirt Johnston, Mississippi; H. H. Mudd, Missouri; J. Black, Nebraska; G. P. Conn, New Hampshire; D. O. English, New Jersy; A. N. Bell, New York; J. C. Walker, North Carolina; J. C. Reeve, Ohio; H. Carpenter, Oregon; Benjamin Lee, Pennsylvania; E. M. Snow, Rhode Island; R. A. Kinloch, South Carolina; Dr. T. A. Atchinson, Tennessee; H. W. Brown, Texas; F. D. Cunningham, Virginia; L. C. Butler, Vermont; E. A. Hildreth, West Virginia; J. T. Reese, Wisconsin; Joseph R. Smith, United States army.

The Committee also reported regulations of printing the proceedings of the Association and the deposit of its funds.

Dr. N. S. Davis, of Chicago, said it gave him peculiar pleasure to offer one resolution. He then read a resolution of thanks in which he expressed the hearty gratitude of the association to the various railroads in the country who had extended favors: To the Ocean Steamship Company of Savannah, to the local press for courteous attention and accurate reports, to the committee of local arrangements, to the medical fraternity of the city, and last, in glowing language, to the people of "the queen city of the empire State of the South," as the resolutions termed Atlanta.

The resolutions were adopted by a unanimous rising vote of the association.

The last business in order was the installment of the new officers.

Dr. Louis A. Sayre, of New York, the President-elect, came forward and Dr. Theophilus Parvin, of Indianapolis, the retiring President, thus addressed the body:

Gentlemen of the American Medical Association: Less than one year ago we met in a great city of the empire State of the North. To day we are to part in a great city of the empire State of the South. Then we stood where we could almost hear the roar of Niagara. To-day we are in this beautiful city with its kindly hearts, its profusion of lovely flowers, its warm welcomes, sweet as the spring time air. Here in the gladness of spring, with its buds and its blossoms, its beautiful emblems speaking to us of life and the resurrection of life. [Applause.] One year ago you honored me. I thank you for it. I hope I have not disgraced that honor, though I am aware I have not fully met the requirements. To-day I resign the chair to one whose name is not only known throughout our own land, but is recognized and honored abroad wherever the fame of American surgery has reached, Dr. Louis A. Sayre, of New York, the President-elect of the Americal Medical Association of America. [Applause.]

Dr. Sayre took the chair, and said: Gentlemen, I cannot fully express to you the sense of my appreciation of the honor you have conferred on me. I think no man can hold a higher or more honorable position than that of President of the American Medical Association. Oh, that I had the tongue of a Parvin or a Grissom that I might speak to you as I feel. But I cannot express that which fills my heart. I thank you for the honor and will perform the duties of my office as best I can." [Applause.]

Dr. Parvin then said to the association: The hour of adjournment is now at hand. I wish you many happy returns of the pleasant occasion which is now passing from us; happy returns to your loved ones at home and long life and prosperity to you all." [Applause.]

The association then adjourned.

After adjournment, Dr. Sayre exhibited his mode of treating spinal troubles, by plaster of Paris jacket with suspension.

And now that our task is done, we cannot close without expressing our thanks to the gentlemanly and courteous Secretary, for kindness shown us in reporting the proceedings of the Medical Association. To Professor Conner, we are greatly obliged for assistance in preparing the report of the College Association, and to Professor Starling Loving, for kindness shown during the session of the Convention of Colleges.

We have attempted to give an epitomised report only. The details will be found in the regular publication of the proceedings.

Our reports of all the meetings are as full and complete as was possible to have them them under the circumstances. With two meetings occurring at the same time, as was often the case, several items may have been overlooked; and the five sections meeting at the same hour made it impossible to give even a synopsis of their reports and discussions. Taken all together, there has never been at one time, so many and important national assemblages of medical men in America before, and perhaps never will be again. The National College Convention met for the first time, and, it may be, the last time, in Atlanta. For exhibition of intellectual endowments, decorum and gentlemanly bearing members of the various bodies were never surpassed by any collection of men in the Gate City. Five hundred of these quietly listening to the President's charming annual address on the 6th, was indeed an imposing spectacle.

